# Inducible expression of (pp)pGpp synthetases in *Staphylococcus aureus* is associated with activation of stress response genes

**DOI:** 10.1371/journal.pgen.1009282

**Published:** 2020-12-30

**Authors:** Petra Horvatek, Andrea Salzer, Andrew Magdy Fekry Hanna, Fabio Lino Gratani, Daniela Keinhörster, Natalya Korn, Marina Borisova, Christoph Mayer, Dominik Rejman, Ulrike Mäder, Christiane Wolz

**Affiliations:** 1 Interfaculty Institute of Microbiology and Infection Medicine, University of Tuebingen, Germany; 2 Quantitative Proteomics & Proteome Center Tuebingen, University of Tuebingen, Germany; 3 Institute of Organic Chemistry and Biochemistry, Czech Academy of Sciences, Prague, Czech Republic; 4 Interfaculty Institute for Genetics and Functional Genomics, University Medicine Greifswald, Greifswald, Germany; University of Wisconsin-Madison, UNITED STATES

## Abstract

The stringent response is characterized by the synthesis of the messenger molecules pppGpp, ppGpp or pGpp (here collectively designated (pp)pGpp). The phenotypic consequences resulting from (pp)pGpp accumulation vary among species and can be mediated by different underlying mechanisms. Most genome-wide analyses have been performed under stress conditions, which often mask the immediate effects of (pp)pGpp-mediated regulatory circuits. In *Staphylococcus aureus*, (pp)pGpp can be synthesized via the RelA-SpoT-homolog, Rel_*Sau*_ upon amino acid limitation or via one of the two small (pp)pGpp synthetases RelP or RelQ upon cell wall stress. We used RNA-Seq to compare the global effects in response to induction of the synthetase of *rel-Syn* (coding for the enzymatic region of Rel_*Sau*_) or *relQ* without the need to apply additional stress conditions. Induction of *rel-Syn* resulted in changes in the nucleotide pool similar to induction of the stringent response via the tRNA synthetase inhibitor mupirocin: a reduction in the GTP pool, an increase in the ATP pool and synthesis of pppGpp, ppGpp and pGpp. Induction of all three enzymes resulted in similar changes in the transcriptome. However, RelQ was less active than Rel-Syn and RelP, indicating strong restriction of its (pp)pGpp-synthesis activity *in vivo*. (pp)pGpp induction resulted in the downregulation of many genes involved in protein and RNA/DNA metabolism. Many of the (pp)pGpp upregulated genes are part of the GTP sensitive CodY regulon and thus likely regulated through lowering of the GTP pool. New CodY independent transcriptional changes were detected including genes involved in the SOS response, iron storage (e.g. *ftnA*, *dps*), oxidative stress response (e.g., *perR*, *katA*, *sodA*) and the *psmα1–4 and psmß1-2* operons coding for cytotoxic, phenol soluble modulins (PSMs). Analyses of the *ftnA*, *dps* and *psm* genes in different regulatory mutants revealed that their (pp)pGpp-dependent regulation can occur independent of the regulators PerR, Fur, SarA or CodY. Moreover, *psm* expression is uncoupled from expression of the quorum sensing system Agr, the main known *psm* activator. The expression of central genes of the oxidative stress response protects the bacteria from anticipated ROS stress derived from PSMs or exogenous sources. Thus, we identified a new link between the stringent response and oxidative stress in *S*. *aureus* that is likely crucial for survival upon phagocytosis.

## Author summary

Most bacteria make use of the second messenger (pp)pGpp to reprogram bacterial metabolism under nutrient-limiting conditions. In the human pathogen *Staphylococcus aureus*, (pp)pGpp plays an important role in virulence, phagosomal escape and antibiotic tolerance. Here, we analyzed the immediate consequences of (pp)pGpp synthesis upon transcriptional induction of the (pp)pGpp-producing enzymes Rel, RelP or RelQ. (pp)pGpp synthesis provokes immediate changes in the nucleotide pool and severely impacts the expression of hundreds of genes. A main consequence of (pp)pGpp synthesis in *S*. *aureus* is the induction of ROS-inducing toxic phenol soluble modulins (PSMs) and simultaneous expression of the detoxifying system to protect the producer. This mechanism is likely of special advantage for the pathogen after phagocytosis.

## Introduction

The stringent response is characterized by the synthesis of the alarmones pGpp, ppGpp and pppGpp, here collectively named (pp)pGpp. (pp)pGpp interferes with many cellular processes, including transcription, replication and translation [1,2,3,4,5,6,7,8,9,10]. Depending on the species, the stringent response is crucial for diverse biological processes, including differentiation, biofilm formation, antibiotic tolerance, production of secondary metabolites or virulence [8,11]. It is now clear that there are fundamental differences between the stringent response initially characterized in *Eschericia coli* and the stringent response in Firmicutes [7,9]. Differences have been observed in the enzymes involved in the synthesis and degradation of the messengers and in the downstream effects of (pp)pGpp.

(pp)pGpp is synthesized by RelA-SpoT-homologs (RSHs) by transferring pyrophosphate originating from ATP to the 3´ OH group of GTP, GDP or GMP. Long RSH enzymes are present in nearly all bacteria and show a conserved molecular architecture composed of a C-terminal sensory region and an N-terminal enzymatic region with distinct (pp)pGpp hydrolase and synthetase domains [12]. Firmicutes, such as *Staphylococcus aureus*, possess one long RSH enzyme, Rel_*Sau*_ and in addition two small alarmone synthetases (SAS), RelP and RelQ. Amino acid limitation is the only condition known to induce a Rel_*Sau*_-mediated stringent response phenotype [13]. Under non-inducing conditions, Rel_*Sau*_ is primarily in a hydrolase-On/synthetase-Off conformation even when the C-terminal sensory region is deleted [14]. The strong hydrolase activity of Rel_*Sau*_ makes the enzyme essential for the detoxification of (pp)pGpp produced by RelP or RelQ [13].

RelP and RelQ are part of the VraRS cell-wall stress regulon [15] and are thus transcriptionally induced, e.g., after vancomycin treatment [16]. Thereby, they contribute to tolerance towards cell-wall active antibiotics such as ampicillin or vancomycin. Recently, structural and mechanistic characterization revealed that RelQ from *Bacillus subtilis* and *Enterococcus faecalis* form tetramers [17,18]. RelQ activity is strongly inhibited through the binding of single-stranded RNA. pppGpp binding leads to disassociation of the RelQ:RNA complex and its activation [18]. In contrast, RelP activity is inhibited by both pppGpp and ppGpp, activated by Zn^2+^ and insensitive to inhibition by RNA [19,20]. For the *S*. *aureus* enzymes it could be shown that RelQ, but not RelP are allosterically stimulated by the addition of pppGpp, ppGpp or pGpp [21]. Thus, although highly homologous, RelP and RelQ seem to have different functions within the cell. One can assume that different post-translational regulatory mechanisms are in play to fine-tune (pp)pGpp synthesis under different growth conditions.

In *S*. *aureus*, the stringent response plays important roles in virulence [13], phagosomal escape [22] and antibiotic tolerance [8,16,23,24,25,26,27]. The enzymes HprT and Gmk involved in GTP synthesis, putative GTPases (RsgA, RbgA, Era, HflX, and ObgE) and DNA primase were identified as (pp)pGpp target proteins [23,28]. (pp)pGpp binding inhibits enzyme activities, resulting in lowering of the GTP pool and inhibition of the translation apparatus and replication. Of note, in contrast to *E*. *coli*, (pp)pGpp from Firmicutes does not interfere with RNA polymerase activity [10]. Instead, in these organisms, (pp)pGpp regulates transcription via an indirect mechanism that strongly relies on the lowering of the intracellular GTP pool [22,28,29,30]. A decrease in the GTP level leads to the repression of nucleotide-sensitive, GTP-initiating promoters, e.g., those of rRNA genes [30,31]. Low GTP levels also affect the CodY regulon. The transcription factor CodY, when loaded with GTP and branched-chain amino acids, acts mainly as a repressor of many genes involved in amino acid synthesis and virulence [32,33]. The global transcriptional effects of (pp)pGpp have been examined previously in several Firmicutes, such as *B*. *subtilis* [34], *Streptococcus pneumoniae* [35], *Enterococcus faecalis* [36], *Streptococcus mutans* [37] and *S*. *aureus* [22]. These studies were based on the comparison of the wild-type and *rel* mutant strains under conditions mimicking amino acid starvation. Of note, these stress conditions are accompanied by profound physiological changes, which are only partially mediated by (pp)pGpp. For instance, amino acid limitation leads to the stabilization of many transcripts independent of (pp)pGpp [13]. Thus, from these analyses, it is difficult to draw firm conclusions on the primary transcriptional changes imposed by (pp)pGpp synthesis. Recently, one study tried to circumvent this drawback by transcriptional induction of (pp)pGpp synthetase in *E*. *coli* and gained major new insights [38].

Here, we aimed to compare the Rel-, RelQ- and RelP-mediated effects on nucleotide pools, transcription and functional consequences without imposing nutrient starvation or stress. Therefore, the Rel synthetase (Rel-Syn, N-Terminal region of Rel with mutated hydrolase), RelP and RelQ were expressed from an anhydrotetracycline (ATc)-inducible promoter in a (pp)pGpp^0^ strain in which the enzymatic domains of all three synthetases were mutated. Through RNA-Seq analyses, we identified new (pp)pGpp-regulated genes, many of which are involved in the oxidative stress response, iron storage and the synthesis of phenol-soluble modulins (PSMs). Thus, (pp)pGpp synthesis contributes not only to PSM-derived ROS production but also to protection from these toxic molecules.

## Results

### Changes in the nucleotide pools after transcriptional induction of *rel-Syn* and *relQ*

We first compared the stringent response imposed by mupirocin (isoleucyl-tRNA synthase inhibitor [39]) with the genetic induction of (pp)pGpp synthetases. To analyse Rel dependent (pp)pGpp synthesis without stress, we cloned the N-terminal region of Rel with inactivated hydrolase domain [14] designated as Rel-Syn. *Rel-Syn* or *relQ* were expressed using an ATc-inducible expression system in a (pp)pGpp^0^ strain background. Strain (pp)pGpp^0^ is unable to synthesize (pp)pGpp due to mutations in all three (pp)pGpp synthetases (full deletion of *rel*, synthetase mutations in *relP* and *relQ* [14]). Strains were grown to an early exponential growth phase and gene expression was induced by ATc for 30 min. Sub-inhibitory concentration of ATc resulted in similar induction of *rel-Syn* or *relQ*, (7.0 and 6,7 log_2_ fold increase compared to un-induced, respectively, S1 Data). ATc treatment did not influence gene expression in strains containing the empty control plasmid pCG248 (S1 Fig).

**Fig 1:**
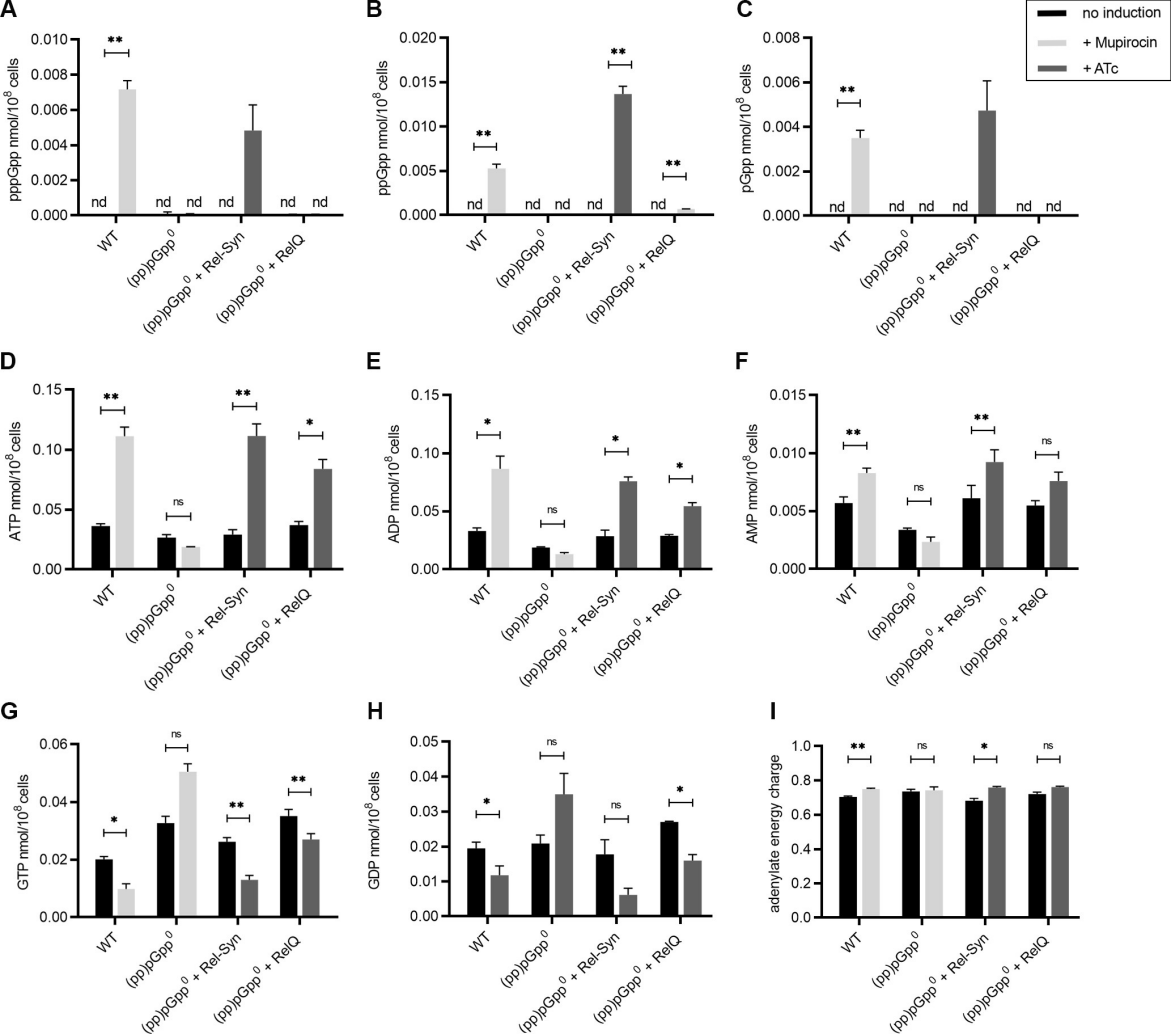
Changes in the nucleotide pool after mupirocin treatment or transcriptional induction of *rel-Syn* or *relQ*. Strain HG001 and derivatives were grown to OD_600_ = 0.3 and treated for 30 min with or without 0.125 μg/ml mupirocin (lightgrey) or 0.1 μg/ml ATc (darkgrey). Nucleotide analyses were performed using mass spectrometry (ESI-TOF) in negative ion mode. Error bars represent SEM (n = 3) from three biological replicates. The adenylate energy charge was calculated by [ATP] + 0.5 [ADP]/[ATP] + [ADP] + [AMP]. Statistical significance was determined by two-tailed Student´s T-test, *p ≤ 0.05, **p ≤ 0.01, ***p ≤ 0.001 and ****p ≤ 0.0001.

Quantification of the nucleotide pools after mupirocin treatment or induction of *rel-syn* or *relQ* revealed that induction of *rel-syn* resulted in similar levels of pppGpp, ppGpp, and pGpp as induction of the stringent response in the wild type by mupirocin (Fig 1). In Firmicutes, (pp)pGpp inhibit several enzymes involved in purine nucleotide synthesis and transport [7]. Accordingly, the concentrations of guanine nucleotides GTP and GDP was negatively correlated to (pp)pGpp synthesis (Fig 1). Of note, (pp)pGpp synthesis leads to concomitant increase of adenine nucleotides (ATP, ADP and AMP) and a slight increase in the adenylate energy charge. In the (pp)pGpp^0^ strain, mupirocin treatment resulted in significant increase of GTP and decrease ATP concordant with previous results [30]. In summary, Rel-mediated (pp)pGpp synthesis imposed by mupirocin or genetic *rel-syn* induction resulted in similar changes of the nucleotide pool characterised by reduction of guanine nucleotides and accumulation of adenine nucleotides. After induction of *relQ*, similar changes in the nucleotide pools were detectable. However, the effect of *relQ* induction on the nucleotide pools was significantly lower than that of *rel-syn* induction or mupirocin treatment. Thus, *rel-syn* induction probably mirrors stress conditions (e.g. upon mupirocin treatment), whereas *relQ* expression more likely reflect basal low level (pp)pGpp synthesis.

### Impact of (pp)pGpp synthesis on the transcriptome

**Fig 2:**
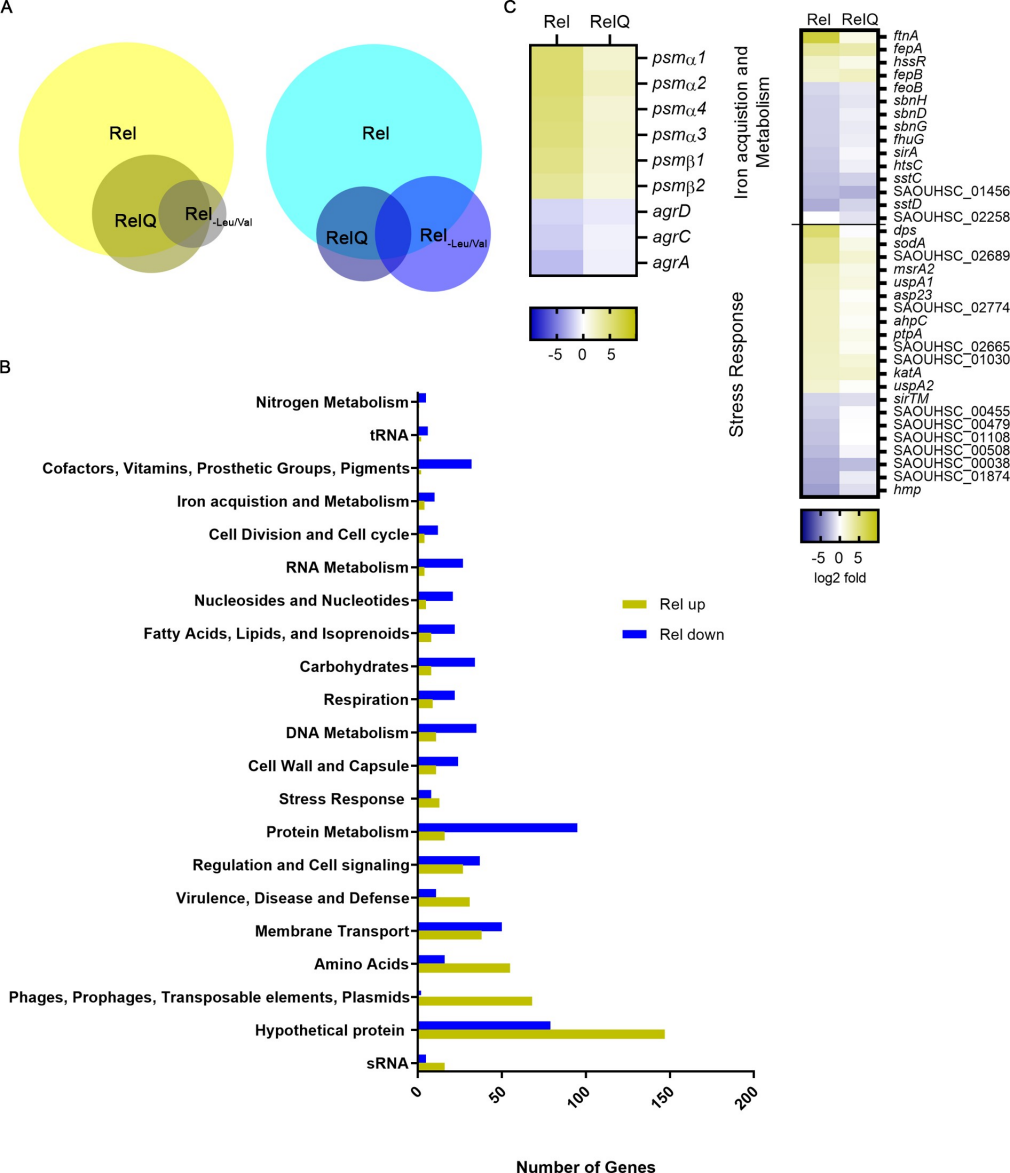
Global changes in gene expression upon transcriptional induction of *rel-Syn* or *relQ*. (pp)pGpp^0^ with inducible *rel-Syn* or *relQ* was grown to OD_600_ = 0.3 and treated for 30 min without or with 0.1 μg/ml ATc. **A**. Venn diagrams showing genes or sRNAs upregulated (yellow) or downregulated (blue) after induction in comparison to uninduced cultures (< 3-fold difference, p< 0.001). Previously, described stringent genes [22] are indicated as Rel_-Leu/Val_. **B**. Genes with significant changes after induction of *rel-syn* (< 3-fold difference, p< 0.001) according to functional categories. **C.** Heatmap representing Rel-Syn-dependent up- and downregulated genes assigned to the functional categories iron acquisition and metabolism, stress response and Agr-related genes.

We next analysed the impact of (pp)pGpp synthesis after induction of *rel-syn* or *relQ* on mRNA abundance. RNA-Seq data revealed that 1074 genes or sRNAs were significantly affected by either Rel-Syn (total: 478 up, 551 down) or RelQ (total: 155 up, 97 down) with a large overlap in genes affected by Rel-Syn and RelQ (Fig 2A and S1 Data). However, consistent with the nucleotide measurements (Fig 1), the effect of *relQ* induction was less prominent (S1 Table). We compared the RNA-Seq data with previous microarray analyses obtained after stringent response imposed by transferring bacteria to amino acid limiting conditions (-Leu, -Val) [22]. Most of the previously identified stringent response genes were confirmed by the RNA-Seq analysis (Fig 2A). Of note, in the present analysis, only genes with at least three-fold differences and a significance level of p < 0.001 were included in the analysis shown in Fig 2 and S2 Data. Despite the higher stringency in the analysis, the present analysis revealed far more (pp)pGpp-regulated genes. Genes were classified into functional categories using the SEED annotation (http://pubseed.theseed.org). (pp)pGpp induction resulted in the downregulation of many genes involved in protein synthesis (e.g ribosomal proteins RplA-T) and RNA/DNA (e.g. purine biosynthesis, gyrase) metabolism consistent with previous results that the stringent response mainly leads to the shutdown of translation and replication [22] (Fig 2B). Many of the genes upregulated by Rel-Syn and RelQ are part of the CodY regulon (S1 Data) and thus likely regulated by lowering of the GTP pool. The sRNA, RsaD was recently described to be directly repressed by CodY [40]. Concordantly, *rsaD* was found strongly up-regulated upon *rel-Syn* and *relQ* induction (8.5 and 6.0 log_2_ fold change, respectively) and used as read-out for a prototypic CodY target is subsequent experiments. *AlsS* was identified as RsaD repressed target gene [40] and as expected downregulated upon *rel-Syn* expression. Thus, the genetic approach proved to gain reliable, physiologically relevant results as validated by comparison with previous studies on (pp)pGpp mediated transcriptional changes. We further focused on so far unknown genes/phenotypes which were induced during stringent conditions. Therefore, we concentrated on genes that were strongly affected by *rel-syn* induction (Fig 2C and S1 Data) but not known to be under the control of CodY. Interestingly, many of these genes were assigned to iron acquisition/metabolism (upregulation of genes involved in iron storage; downregulation of genes involved in siderophore biosynthesis and iron transport), stress response (*dps*, *sodA*, *katA ahpC*, *uspA1/2*, *asp23*, *ptpA*, and *msrA2)*, and virulence (upregulation of *psmsα/ß*; downregulation of *agr*). Phage-encoded genes were also upregulated, indicating phage-inducing conditions. This is in line with the upregulation of *recA* and *lexA*. For further analysis, we selected *ftnA*, *dps*, *agr* and *psm*α as read-out for (pp)pGpp-mediated CodY independent activities.

### Comparison of the mupirocin-induced stringent response and induction of *rel-Syn*

**Fig 3:**
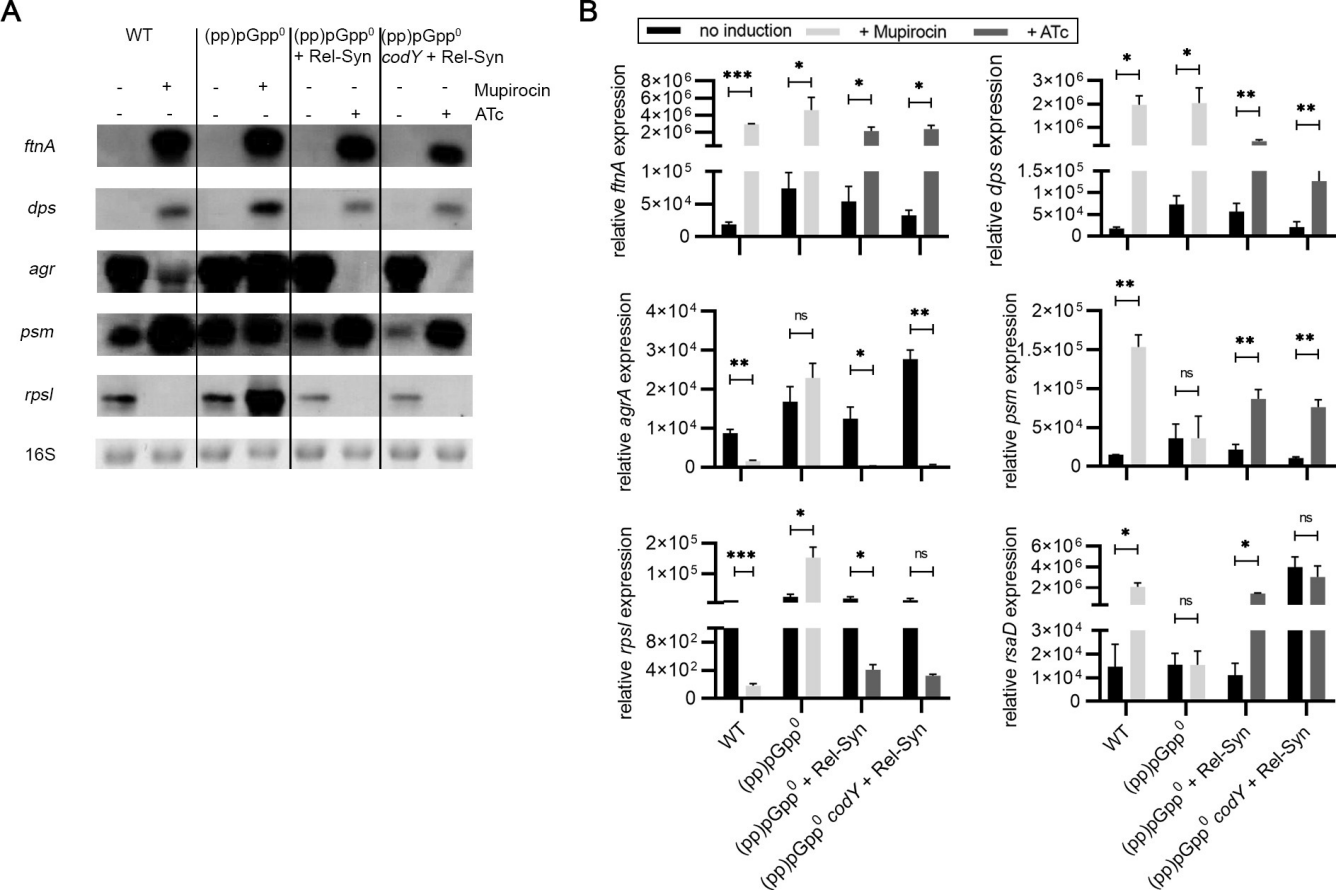
Correlation of mupirocin-induced stringent response and transcriptional induced *rel-syn* for selected CodY-independent genes. Strain HG001 and derivatives were grown to OD_600_ = 0.3 and treated for 30 min with or without 0.125 μg/ml mupirocin or 0.1 μg/ml ATc (mutant strains with inducible *rel-Syn*). **A**. For Northern blot analysis, RNA was hybridized with digoxigenin-labelled probes specific for *ftnA*, *dps*, *psm*, *agrA or rpsl*. The 16S rRNA detected in ethidium bromide-stained gels is indicated as a loading control in the bottom lane. **B.** Quantification of mRNA by qRT-PCR based on three biological replicates. Statistical significance was determined by two-tailed Student´s T-test, *p ≤ 0.05, **p ≤ 0.01, ***p ≤ 0.001 and ****p ≤ 0.0001.

We compared the expression of the selected genes after induction of the stringent response via mupirocin and after *rel-Syn* induction by Northern blot analysis (Fig 3A) and qRT-PCR (Fig 3B). We verified the upregulation of *ftnA*, *dps*, *psm* and the sRNA *rsaD* under both conditions. Mupirocin also resulted in *ftnA* and *dps* activation in the (pp)pGpp^0^ strain indicating additional (pp)pGpp-independent effects of mupirocin on the expression of these genes. (pp)pGpp-mediated activation of *psm* expression is clearly not correlated to *agr* expression. Agr is the main activator required for *psms* expression [41]. Notably, the expression of the *agr* operon was even lower upon (pp)pGpp synthesis (S1 Data and Fig 3A and 3B), indicating that (pp)pGpp induces *psm* expression independent of Agr. Induction of *rel-Syn* in wild type background resulted in minor changes in gene expression compared to induction in the (pp)pGpp^0^ strain (S2 Fig). This emphasizes the strong hydrolase activity of Rel as present in the wild type leading to rapid hydrolysis of (pp)pGpp [14].

### CodY-independent activation of gene expression upon *rel-Syn* induction

(pp)pGpp synthesis leads to the lowering of the GTP pool and subsequently to de-repression of CodY target genes. Indeed, many of the genes that were upregulated in response to *rel-Syn* induction belong to the CodY regulon (S1 Data). However, the selected marker genes are not supposed to be regulated via CodY. For further validation we induced *rel-Syn* in a *codY* negative (pp)pGpp^0^ strain (Fig 3). The CodY target *rsaD* was confirmed to be de-repressed in the *codY* negative background [40]. However, other selected genes showed similar expression patterns in *codY*-positive and *codY*-negative backgrounds (Fig 3A and 3B). Thus, (pp)pGpp impacts the expression of these genes independent of CodY.

### Induction of *rel-Syn*, *relQ* and *relP* in *S. aureus* USA300

**Fig 4:**
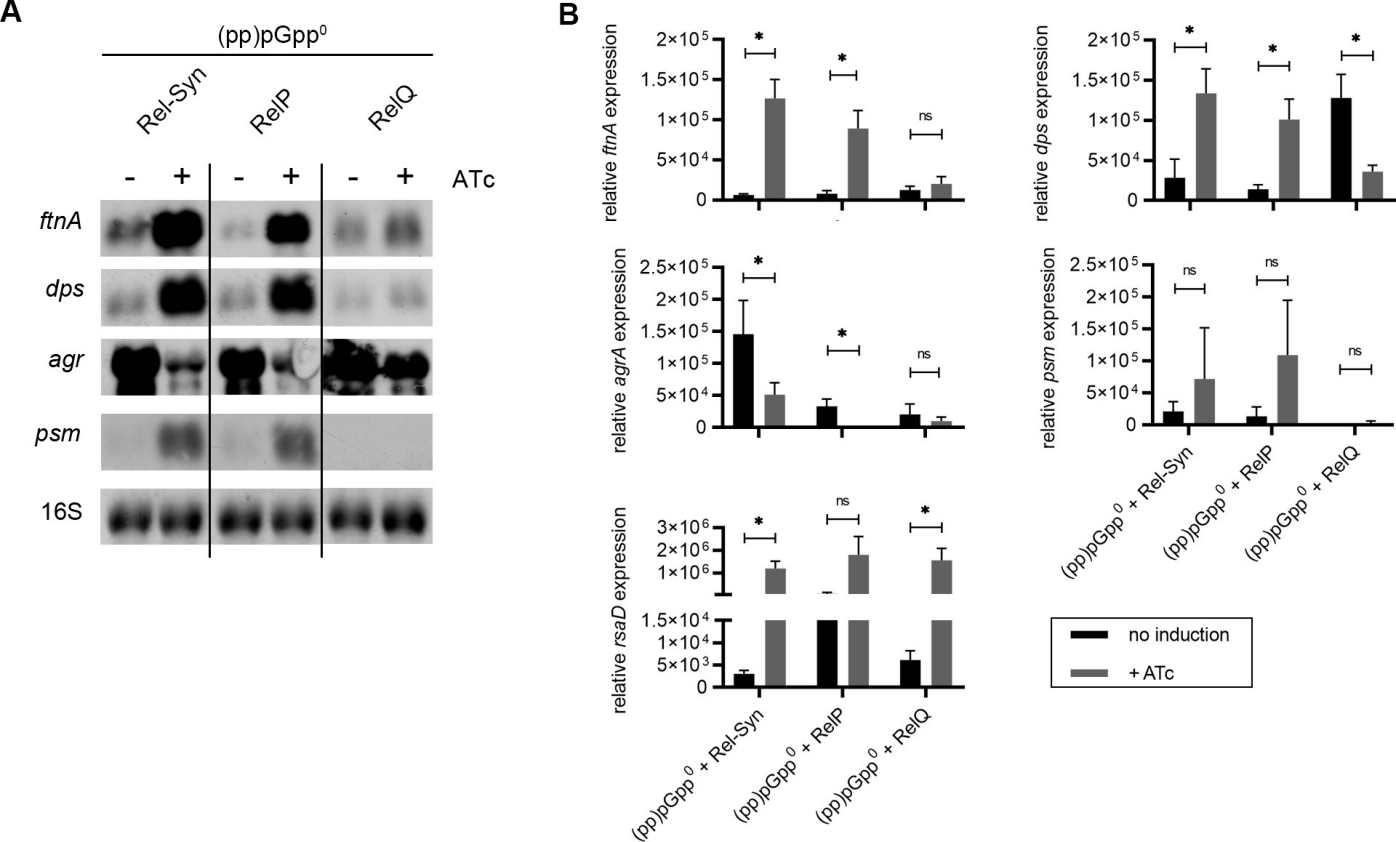
Gene expression following induction of *rel-Syn*, *relQ* or *relP* in USA300 strain background. **A.** Strain USA300 and derivatives were grown to OD_600_ = 0.3 and treated for 30 min without or with 0.1 μg/ml ATc (mutant strains with inducible *rel-Syn*, *relP* or *relQ*). For Northern blot analysis, RNA was hybridized with digoxigenin-labelled probes specific for *ftnA*, *dps*, *psmα* or *agrA*. The 16S rRNA detected in ethidium bromide-stained gels is indicated as a loading control in the bottom lane. **B**. Quantification of mRNA by qRT-PCR based on three biological replicates. Statistical significance was determined by two-tailed Student´s T-test, *p ≤ 0.05, **p ≤ 0.01, ***p ≤ 0.001 and ****p ≤ 0.0001.

To confirm that the observed gene expression pattern is not restricted to strain HG001 and not due to potential second site mutations in the (pp)pGpp^0^ strain we repeated key experiments in strain USA300. Therefore a (pp)pGpp^0^ strain was constructed by sequential mutation of *relP*, *relQ* and *rel*. *rel-Syn*, *relQ* and *relP* were induced from the ATc inducible expression vectors as described. The transcriptional changes imposed by *rel-Syn* induction recapitulated the findings of *rel-Syn* induction in strain HG001 (Fig 4). We also verified that induction of *relQ* only slightly effects marker gene expression (Fig 4). However, induction of *relP* was highly effective, resulting in an expression pattern comparable to induction of *rel-Syn*. The strong effect of *relP* induction could be due to the fact that in contrast to *relP* it does not require pppGpp activation [20]

### *Rel-Syn* induction influences the oxidative stress response and virulence independent of PerR, Fur or SarA

**Fig 5:**
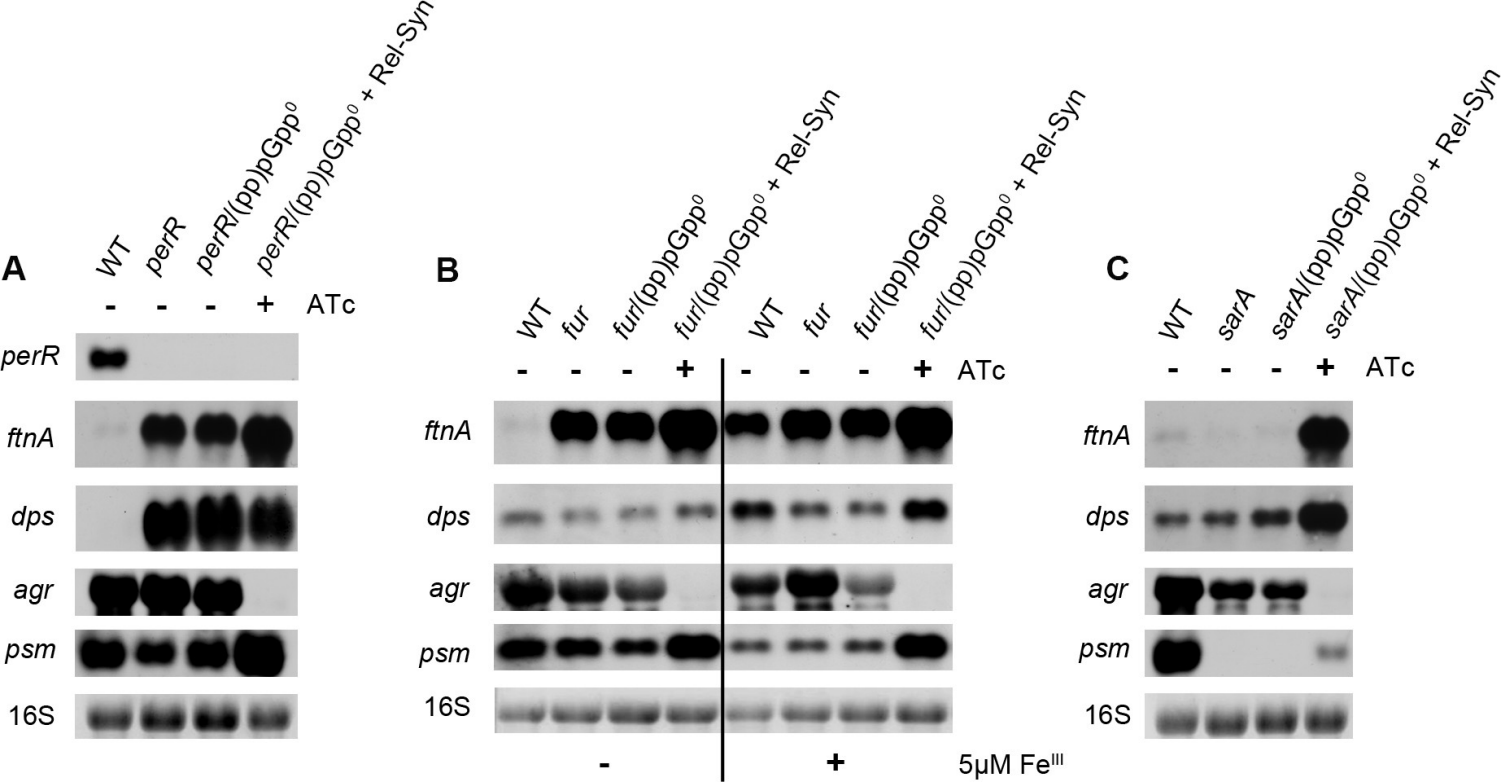
(pp)pGpp-dependent transcriptional changes independent of PerR, Fur or SarA. Strain HG001 and derivatives were grown to OD_600_ = 0.3 and treated for 30 min without or with 0.1 μg/ml ATc (mutant strains with inducible *rel-Syn*). For Northern blot analysis, RNA was hybridized with digoxigenin-labelled probes specific *for ftnA*, *dps*, *psmα* or *agrA*. The 16S rRNA detected in ethidium bromide-stained gels is indicated as a loading control in the bottom lane. For quantitative results see S3 Fig.

Some of the prominent (pp)pGpp-activated genes are known to be under the control of other global regulators, such as PerR, Fur and SarA [42]. We speculated that (pp)pGpp dependent gene expression may somehow be mediated via these global regulators. Therefore, we analysed *rel-Syn* induction in *per*, *fur* and *sarA* mutants (Figs 5A and S3A).

The (pp)pGpp controlled genes *ftnA*, *dps*, *ahpC*, *katA* and *perR* (S1 Data*)* are likely controlled via binding of PerR to a conserved PerR-binding motif (based on the public databases RegPrecise [43] and Aureowiki [44]). As expected, *ftnA* and *dps* were both upregulated in the *perR* mutants. Inducing *rel-Syn* in *perR*/(pp)pGpp^0^ strain showed further increase in *ftnA*, supporting that (pp)pGpp acts in addition and independent of PerR. For *dps*, the *perR* mutation also resulted in high expression, which was slightly decreased by (pp)pGpp. *PerR* deletion resulted in a slight decrease in *psm* expression, which was compensated by *rel-syn* induction. Thus, (pp)pGpp also affects gene expression in a *per*-negative background.

We found that many of the genes affected by Rel-Syn are indicative of iron overload conditions (e.g. upregulation of *ftnA* and *dps (*Fig 2D). We induced *rel-Syn* in a *fur*/(pp)pGpp^0^ background under low and high iron conditions (Figs 5B and S3). Independent of the availability of iron, *ftnA*, *dps* and *psm* were upregulated and *agr* was downregulated after *rel-Syn* induction in the *fur*-negative background indicating that (pp)pGpp regulation is not determined by iron availability or *fur* regulation.

SarA was shown to activate transcription of the *agr* operon [45,46] and proposed to be involved in oxidative stress sensing via a single Cys9 residue [47,48,49]. *sarA* was found to be significantly upregulated by Rel-Syn (S1 Data). *ftnA* and *dps* expression was not influenced by *sarA* mutation (Figs 5C and S3). *agr* and *psm* expression was downregulated in the *sarA* mutant, consistent with the proposed activation of the *agr* system by SarA [45]. Induction of *rel-syn* in the *sarA* mutant again showed the typical induction of *ftnA*, *dps* and repression of *agr*. Interestingly, *psm* expression remained hardly detectable in the *SarA* mutant. In the *sarA* mutant the very low Agr activity is likely not sufficient to allow for *psm* expression. Taken together, the results do not support the hypothesis that any of the candidate regulators function as a central hub for the observed (pp)pGpp dependent gene alterations.

### (pp)pGpp is involved in oxidative stress resistance

**Fig 6:**
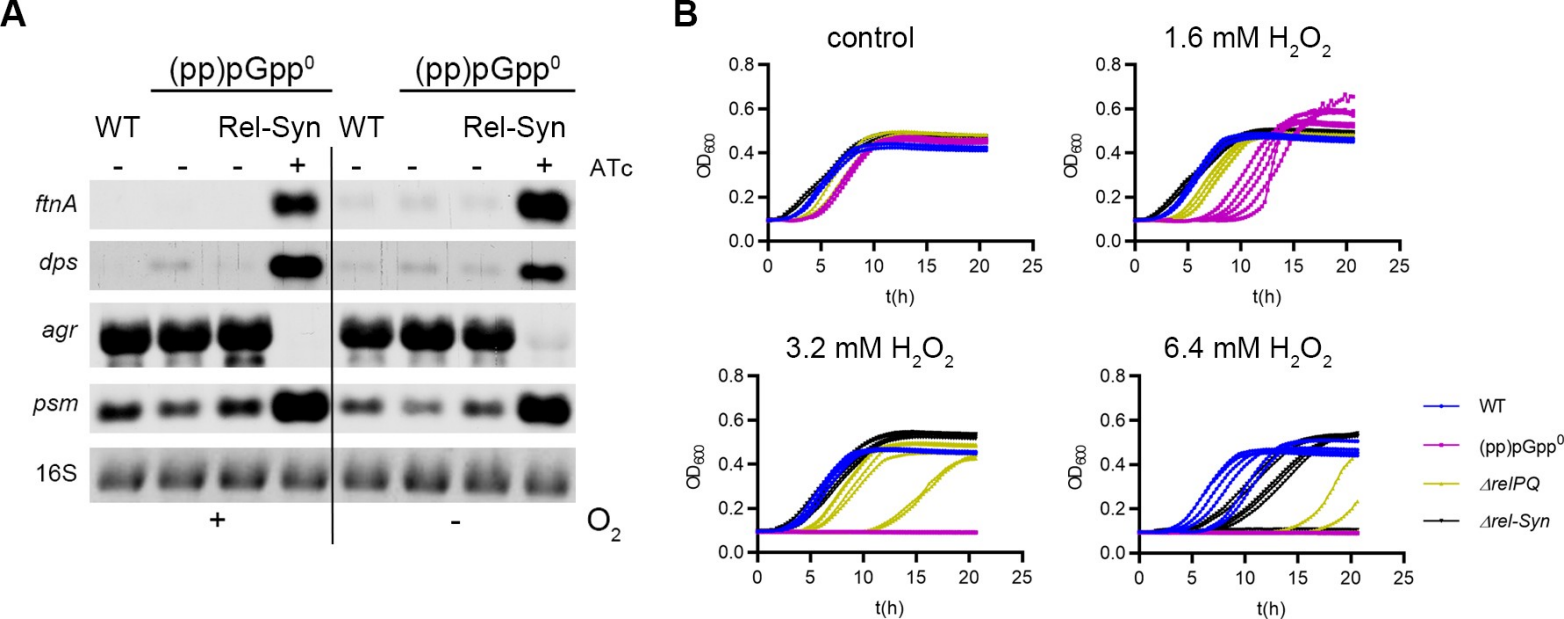
Functional link between stringent response and oxidative stress. **A**. Strain HG001 and derivatives were grown with shaking aerobically or anaerobically to OD_600_ = 0.3 and treated for 30 min without or with 0.1 μg/ml ATc (mutant with inducible *rel-Syn*). For Northern blot analysis, RNA was hybridized with digoxigenin-labelled probes specific for *ftnA*, *dps*, *psm* or *agrA*. The 16S rRNA detected in ethidium bromide-stained gels is indicated as a loading control in the bottom lane. **B** WT, (pp)pGpp^0^, Δ*relPQ* and *rel-Syn* mutants were diluted from overnight culture to an OD = 0.1, challenged with different H_2_O_2_ concentrations, and growth was monitored over time.

Recently, it was shown that PSMs lead to the production of reactive oxygen species (ROS) [50]. One might speculate that under stringent conditions PSM-mediated ROS production triggers the expression of the oxidative stress genes. In this case, induction of *rel-Syn* should not result in the induction of these genes under anaerobic conditions, where ROS cannot be produced. However, *rel-Syn* induction resulted in the same transcriptional pattern regardless of whether bacteria were grown with or without oxygen (Fig 6A). We also analysed whether ROS would result in activation of the stringent response. However, H_2_O_2_ treatment did not affect transcription of stringent response genes such as *fntA*, *dps*, *psm* or *rpsl* (S5 Fig). Thus, (pp)pGpp-mediated gene alterations of the selected marker genes are not a consequence of ROS formation.

These data indicate that (pp)pGpp simultaneously activates ROS-producing PSMs as well as ROS defence systems to prepare cells to withstand oxidative stress. To verify this hypothesis, we challenged wild type and mutant strains deficient in (pp)pGpp synthesis with H_2_O_2_. The (pp)pGpp^0^ strain was indeed more sensitive to oxidative stress. The minimal inhibitor concentration (MIC) of H_2_O_2_ to inhibit growth of the wild type was 6.4 mM and 3.2 mM for the (pp)pGpp^0^ strain. Moreover, the (pp)pGpp^0^ strain was more efficiently killed after 1 h or 2 h incubation with H_2_0_2_ (S4 Fig). Under the assay conditions, the basal (pp)pGpp might be derived from any of the (pp)pGpp synthetases. We analyzed a *relPQ* mutant and a *rel-Syn* mutant in which the synthetase domain of Rel was mutated. Both strains showed an intermittent phenotype in which the MIC varied between 3.2 and 6.4 mM when biological replicates were analyzed. To follow up on these ambiguities, we monitored growth after addition of H_2_O_2_ (Fig 6C). There was high variation in the lag time between biological replicates. Replicates of the *relPQ* or *rel-Syn* mutant showed a delayed lag phase, and some of the replicates could not grow. In cases where no growth was detectable the cultures were sterile as determined by colony counting. The delay of the lag phase was more prominent for the *relPQ* mutant than for the *rel-Syn* mutant. Nevertheless, none of the (pp)pGpp^0^ replicates could resume growth, and this result was consistent with the reproducible lowered MIC of this strain, indicating that (pp)pGpp indeed protects against oxidative stress.

We next speculated that under H_2_0_2_ conditions may induce stringent response in wild type bacteria. However, treatment of bacteria with H_2_O_2_ even at MIC concentrations did not induce stringent marker genes (S5 Fig). This indicates that the basal level of (pp)pGpp produced in wild type bacteria is sufficient to confer resistance.

## Discussion

**Fig 7:**
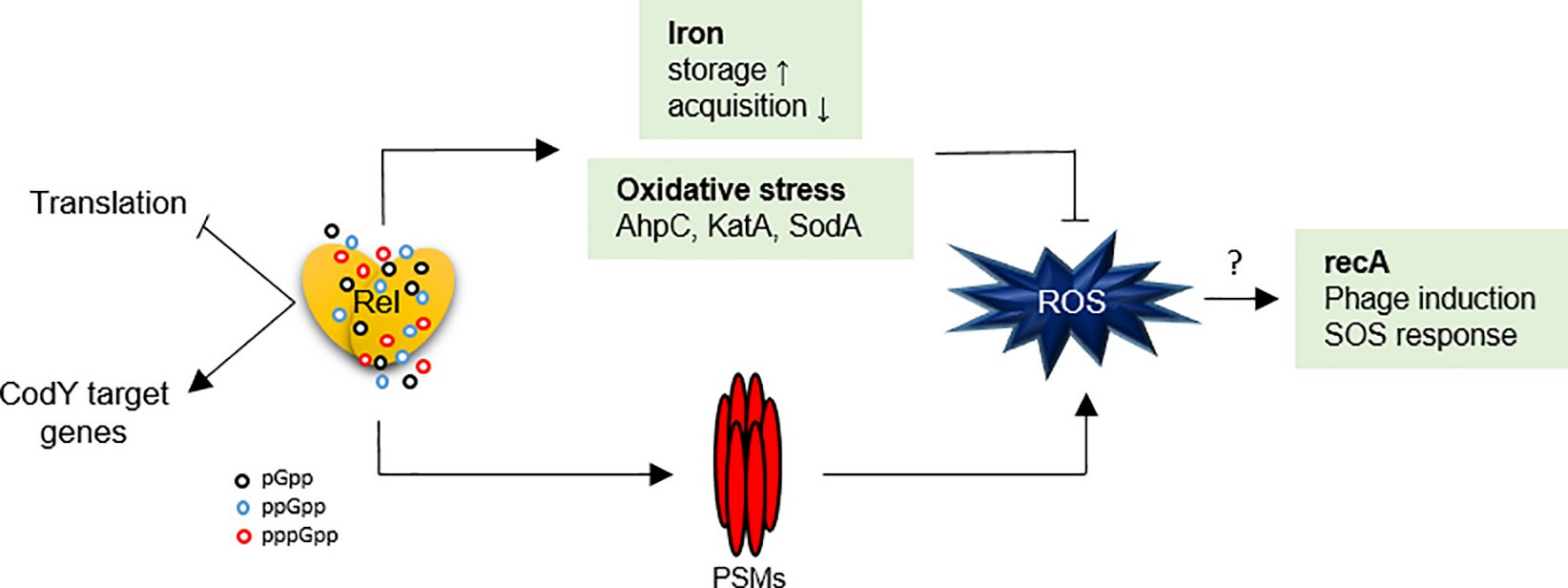
(pp)pGpp protects from oxidative stress. (pp)pGpp leads to upregulation of oxidative stress and iron storage genes. These upregulated genes are beneficial to counteract endogenous (PSMs) or exogenous (e.g., H_2_O_2_) ROS. Upregulation of SOS and phage genes might be a consequence of ROS accumulation, e.g., by PSMs.

We chose a genetic approach to define the early transcriptional response upon (pp)pGpp synthesis without the need to apply additional stress conditions. As expected from previous studies, [22,51] (pp)pGpp synthesis resulted in severe downregulation of the translational machinery and de-repression of CodY target genes. Many additional (pp)pGpp-regulated genes that are presumably important for the survival of *S*. *aureus* during starvation conditions were identified. Regulation of these genes might occur indirectly through other so far ill-defined regulatory circuits or via changes in the nucleotide pool. Here, we focused mainly on genes that were found to be activated upon (pp)pGpp synthesis in a CodY-independent manner, particularly *psm*, *ftnA* and *dps*. The (pp)pGpp-dependent activation of these genes can also occur in strains missing the prototypic proteinaceous transcriptional regulators PerR, Fur, or SarA. These regulators are well known to be involved in the regulation of the selected genes. However, they need to be activated through oxidative stress and/or iron [42]. The RNAseq data revealed that transcription of the repressor *perR* is also upregulated upon (pp)pGpp synthesis indicating a feedback regulation to dampen the response. In summary, (pp)pGpp functions as a complementary, immediate message, allowing cells to react to adverse conditions such as amino acid starvation or cell wall stress. Under these conditions, upcoming oxidative stress seems to be anticipated, and (pp)pGpp prepares the cells for survival, e.g. ROS challenges (Fig 7). Indeed, a (pp)pGpp^0^ strain is more sensitive to H_2_O_2._ Both Rel and RelP/RelQ contribute to the protective effect.

### (pp)pGpp leads to *psm* activation

One of the most prominent effects of (pp)pGpp synthesis is the upregulation of *psmα* and *psmβ*, confirming previous results [22,52]. PSMs are a family of amphipathic, alpha-helical peptides that have multiple roles in staphylococcal pathogenesis and contribute to a large extent to the pathogenic success of virulent staphylococci [53,54]. (pp)pGpp-dependent *psm* expression within neutrophils was shown to be crucial for survival after phagocytosis [22]. However, PSMs also interact with the membrane of the producer, promote the release of membrane vesicles from the cytoplasmic membrane via an increase in membrane fluidity [55,56], reduce persister formation [57,58] and are involved in self-toxicity via ROS formation [50]. Interestingly, (pp)pGpp-dependent *psm* activation was not correlated with the activation of the quorum sensing Agr system, the main regulator required for *psm* expression [41]. Agr was even repressed under (pp)pGpp-inducing conditions. Previously, analysis of a clinical isolate overproducing (pp)pGpp also indicated that (pp)pGpp leads to *agr* inhibition [24]. Thus, (pp)pGpp-mediated *psm* activation is clearly uncoupled from *agr* expression. Recently, the sRNA Teg41 (S131) [59] and the transcriptional regulator MgrA [60], Rsp [61] or Rbf [62] were found to interfere with *psm* expression. However, it is unlikely that they mediate the (pp)pGpp regulatory effect because transcription of these regulators was unaltered based on our RNA-Seq analysis (S1 Data). Thus, the molecular mechanism by which (pp)pGpp leads to *psm* activation has to be elucidated. It is likely that the accompanying changes in the ATP/GTP ratios are crucial for this activation pattern. *psm* promoters might be sensitive to the concentration of the initiating nucleoside triphosphate (iNTP). The +1 position (e.g., G or A) of sensitive genes dictates whether transcriptional initiation/elongation requires high GTP or ATP levels, respectively [29]. Various sequence combinations determine whether a promoter is sensitive to iNTP [63]. Such sequence motifs are hard to predict within *psm* promoters. However, both the p*smα* and *psmß* operons start with an A at the +1 position [41], which might explain the higher expression due to the increased ATP levels following (p)ppGpp synthesis.

### (pp)pGpp and oxidative stress response

Genes whose expression is indicative of iron overload conditions were also highly affected by (pp)pGpp. We recently, could confirm that the (pp)pGpp^0^ strain of strain USA300 has elevated free iron levels contributing to oxidative stress and increased ROS production [64]. A similar effect was reported for *Vibrio cholera* [65]. Here, the expression of the iron transporter FbpA was repressed via (pp)pGpp, resulting in a reduction of intracellular free iron required for the ROS-generating Fenton reaction. This contributed to reducing antibiotic-induced oxidative stress and thus tolerance, and this is also true for *S. aureus* [64]. In addition to interfering with iron metabolism, other genes involved in oxidative stress were activated by (pp)pGpp. A link between the stringent response and oxidative stress response has been observed in different organisms, although the underlying mechanisms and outcome might be highly diverse. (pp)pGpp-dependent upregulation of superoxide dismutase (SOD) was described in *B*. *suis* [66] and *P*. *aeruginosa* [67]. SOD was shown to be the key factor responsible for (pp)pGpp-mediated multidrug tolerance in *P*. *aeruginosa* [67]. Moreover, (pp)pGpp-deficient strains are often found to be more sensitive to oxidative stress [68,69,70]. However, in *E*. *faecalis*, a (pp)pGpp^0^ strain grew faster and to a higher growth yield than its parent in the presence of H_2_O_2_ [71].

Here, we show that the stringent response in *S*. *aureus* leads to the activation of ROS-inducing toxins and simultaneous expression of the detoxifying system to protect the producer. This is likely a special advantage for the pathogen once it encounters neutrophils and elevated ROS. (pp)pGpp-dependent PSM synthesis is required to escape from within cells after phagocytosis [22,72]. The upregulation of the oxygen stress programme will help protect the cell from endogenous as well as exogenous ROS.

### Comparison of Rel-Syn, RelQ and RelP activity

We compared the *in vivo* activity of Rel-Syn, RelQ and RelP. RelP showed similar effect on gene expression as Rel-Syn, whereas RelQ was far less active (Fig 4). Thus, under our growth conditions RelQ activity seems to be restricted *in vivo*. Comparison of RelP and RelQ from other organisms revealed that RelQ is inhibited through RNA binding and auto-activated by (pp)pGpp [19,20]. We analyzed RelQ activity in an (pp)pGpp background under non-stress conditions where RelQ activity is likely restricted via RNA binding and/or the missing basal (pp)pGpp provided by other synthetases. The conditions that would relieve this restriction remain to be determined. A recent biochemical analysis of purified RelP and RelQ from *S*. *aureus* confirmed that RelQ, but not RelP, was allosterically-stimulated by the addition of pppGpp, ppGpp or pGpp [21]. Moreover, in vitro both enzymes were shown to efficiently synthesize pGpp in addition to pppGpp and ppGpp. In our *in vivo* analysis we could detect considerable quantities of pGpp derived from Rel activity (Fig 1). However, *in vitro* Rel seems to preferentially synthesize pppGpp [21]. One may speculate that pGpp detected *in vivo* might be enzymatically derived from NuDiX hydrolase, NahA, an enzyme that in *B*. *subtilis* efficiently produces pGpp by hydrolyzing (p)ppGpp [73]. In this organism, pGpp potently regulates the purine biosynthesis pathway but in contrast to (p)ppGpp does not interact with the GTPases. It was proposed that the different nucleotides may fulfill different roles through fine-tuning in signal transduction. Moreover, the different levels of (pp)pGpp in the cell may also dictate different outcomes. There is now growing evidence that basal level of (pp)pGpp (often below detection limit) are involved to maintain balanced growth whereas high levels are indicative for stress conditions resulting in severe reprogramming of the cell and growth arrest [74,75]. In this context, the induction of *rel-Syn* likely mirrors stress conditions, whereas *relQ* induction with low detectable (pp)pGpp synthesis more resembles the proposed basal level. From the RNA-Seq analysis we found no clear quantitative difference between both conditions. However, many of the genes affected by RelQ could be assigned to the CodY regulon. This illustrates the high sensitivity of CodY towards subtle changes of the GTP pool imposed by (pp)pGpp synthesis.

### Different (pp)pGpp mode of actions result in similar outcome

Recently, genome-wide direct effects on transcription from ppGpp binding to its two sites on RNA polymerase were assessed in *E*. *coli* [38]. Similar to our approach, ppGpp was produced without concurrent starvation by conditional expression of a RelA variant lacking its autoinhibitory domain. The Rel variant was induced for 5–10 min in strains with or without the two binding sites for ppGpp on RNAP. It could be shown that transcriptional changes are in large part due to ppGpp-RNAP interference. However, (pp)pGpp does not bind to RNAP in Firmicutes. We anticipated that for full stringent response in *S*. *aureus* significant changes of the nucleotide pool have to occur and that transcriptional changes are not directly linked to (pp)pGpp in this organism. Therefore, we analysed transcriptional changes after 30 min of *rel-Syn* induction to allow the adjustment of the nucleotide pool. We assume that many transcriptional changes are caused by the observed changes in the nucleotide pool. Alternatively, at least some of them might be linked to secondary effects such as inhibition of growth or translation. In the future, time course experiments and concurrent proteome analyses will help to further clarify this issue. Nevertheless, despite major difference in the experimental set-up and the different underlying regulatory mechanisms between *E*. *coli* and *S*. *aureus* both approaches revealed a surprisingly similar outcome: First, in both organism the genetic approaches revealed many more (pp)pGpp-regulated genes in comparison to traditional analyses applying concurrent starvation. Second, observed (pp)pGpp-mediated transcriptional changes are highly similar: i.) large number of genes related to nucleotide, protein, and RNA metabolism, translation, and DNA synthesis are negatively regulated by ppGpp; ii) amino acid biosynthesis genes are highly upregulated and iii.) genes for the responses to DNA damage, general stress and oxidants responded to ppGpp in both organisms. Thus, stringent response has evolved in different organisms to fulfil similar functions by use of highly different mechanisms.

## Materials and methods

### Strains and growth conditions

Strains and plasmids are listed in S1 Table. For strains carrying a resistance gene a concentration of 10 μg/ml chloramphenicol, 10 μg/ml erythromycin or 100 μg/ml ampicillin was used only for overnight cultures. *S*. *aureus* strains were grown overnight in chemical defined medium (CDM) [32], diluted to an optical density (OD_600_) of 0.05 and grown until the early exponential phase OD_600_ = 0.3 with shaking (220 rpm, 37°C). Gene expression in strains carrying a plasmid with an ATc-inducible promoter was induced at OD_600_ = 0.3 with 0.1 μg/ml ATc for 30 min. We chose 30 min induction to allow adjustment of the anticipated changes of the nucleotide pool. For anaerobic growth the strains were diluted to an OD_600_ = 0.05 in hungate tubes (Chemglass), completely filled with CDM. ATc was applied using a syringe at OD_600_ = 0. 3. For OD measurements and RNA isolation, aliquots were drawn with a syringe.

### Generation of (pp)pGpp^0^ mutant in USA300 JE2

For the USA300 (pp)pGpp^0^ mutant (USA300-229-230-263), lysates were prepared from RN4220 strains containing the mutagenesis vectors pCG229, pCG230 and pCG263, respectively (S1 Table). After plasmid transduction of USA300 JE2, mutagenesis was performed as previously described [76]. To avoid toxic accumulation of (pp)pGpp the genes were mutated in the order *relP*, *relQ* and finally *rel*. Mutations were verified by PCR using oligonucleotides listed in S2 Table.

### Generation of *perR*, *fur*, *sarA* and *psm*α/β (pp)pGpp^0^ mutants in HG001

Φ11 lysates were generated from transposon mutants NE665 (*perR*), NE99 (*fur*) and NE1193 (*sarA*) from the NARSA transposon library [77] to transduce *S*. *aureus* strains HG001 and (pp)pGpp^0^. *psm*α or *psmβ* mutations were transduced using Φ11 lysates from previously described mutants [22]. All transductants were verified by PCR using oligonucleotides listed in S2 Table.

### RNA isolation, Northern Blot analysis and qRT-PCR analysis

RNA isolation and Northern blot analysis were performed as described previously [78]. Briefly, bacteria were pelleted and resuspended in 1 ml TRIzol (Thermo Fisher Scientific) and lysed using zirconia/silica beads (0.1mm diameter) and a high speed homogenizer. RNA was isolated following the recommended procedure by TRIzol manufacturer. For RNA-Seq analysis RNA from the aqueous phase was further purified following the RNA-isolation protocol by Amp Tech ExpressArt RNA ready. Transcripts on the Northern blot were detected by dioxigenin-labeled probes, which were generated by a DNA-labelling PCR-Kit (Roche Life Science). Relative quantification of *ftnA*, *dps*, *agrA*, *α-type psms*, *rsaD* and *rpsl* transcripts by qRT-PCR was performed using the Quantstudio3 (Applied Biosystems) and the QuantiFast SYBR Green RT-PCR Kit (Qiagen). Briefly, 5 μg of total RNA were DNase-treated and diluted 1:10 for qRT-PCR. Relative quantities of transcripts were calculated by a standard curve for each gene generated using 6-fold serial dilution of HG001 wild type RNA. Primers for qRT-PCR are listed inS2 Table.

### *In vivo* nucleotide extraction

Nucleotides were isolated based on published protocol [79]. Briefly, strains were grown in CDM overnight, diluted to an OD_600_ = 0.05 and grown in CDM until an OD_600_ = 0.3. Strains were split and treated with or without 0.1 μg/ml ATc for 30 min at 37°C and 220 rpm shaking. 100 ml bacterial cultures were harvested and transferred into 50 ml centrifuge tubes half filled with ice and centrifuged (5 min, 5000 x g, 4°C). Pellets were immediately frozen in liquid nitrogen and stored at -80°C until usage. Samples were thawed on ice and resuspended in 2M formic acid and incubated for 30 min. Resuspended bacteria were lysed by high speed homogenizer using zirconia/silicia beads (0.1 mm diameter) and kept on ice for 30 min. The aqueous phase was collected and mixed with 3 ml 50 mM NH_4_OAc (pH 4.5), loaded on columns (OASIS Wax cartridge 3xcc) and centrifuged (5000 x g, 5min, 4°C). Columns were pre-treated first with pure 3 ml methanol and then with 3 ml 50 mM NH_4_OAc (pH 4.5). Samples were washed first with 3 ml 50 mM NH_4_OAc (pH 4.5) followed by a washing step with 3 ml methanol. Elution was performed with 1 ml of 20% methanol, 10% NH_4_OH. Eluted nucleotides were flash frozen in liquid nitrogen and lyophilized overnight. Lyophilized nucleotides were resuspended in 100 μl ddH_2_O and analyzed via HPLC-MS. pppGpp and ppGpp standard molecule were purchased Jena Biosciences. pGpp was synthesized starting from conveniently protected guanosine and employing both phosphoramidite and phosphotriester methods (S1 Methods).

### *In vivo* and *in vitro* analysis of (pp)pGpp via HPLC-MS

Nucleotide quantification was performed as described [14]. Briefly, nucleotides were analyzed using ESI-TOF (micrO-TOF II, Bruker) mass spectrometer connected to an UltiMate 3000 High-Performance Liquid Chromatography (HPLC). 5 μl of standards or samples were injected onto SEQuant ZIC-pHILIC column (Merck, PEEK 150 x 2.1 mm, 5 μm). MS analysis was performed in negative-ion mode over the mass range from 200 to 1,000 m/z. MS calibration was done by using a sodium formate solution as the tune mix. Nucleotide standards of AMP (346.0552 m/z), ATP (426.0215 m/z), GTP (521.9828 m/z), pGpp (521.9828m/z), ppGpp (601.9491 m/z) and pppGpp (681.9155 m/z) were diluted 10 times 1:1 from 1 mM until a concentration of 1.95 μM and analyzed by HPLC-MS as previously described [14]. Extracted ion chromatogram (EIC) spectra of all standards were presented in DataAnalysis (Bruker) and the area under the curve (AUC) of the respective EICs was calculated in GraphPad Prism 5 (baseline was set to 150). The obtained AUC values of the diluted standards were used to generate a calibration curve. For absolute nucleotide quantification, the AUC of the samples was plugged into the AUC values of the calibration curve and the concentration of the respective nucleotides in the samples was determined. Nucleotide identification was verified by matching the retention times and m/z values of detected peaks in the samples to the measured nucleotide standards. To separate pGpp from GTP (S6 Fig) we used an expectation–maximization (EM). The relative amount of the first chemical component in the mixture is calculated as $I_{1}=\sum_{k:\mu_{k}\leq t_{c}}\lambda_{k}$. The second component was expressed as *I*_2_ = 1−*I*_1_.

The obtained concentrations of the adenosine nucleotides ATP, ADP and AMP in each sample were used to calculate the adenylate energy charge as described [80].

### H_2_O_2_ killing assay

Strains were grown over night in CDM, diluted in fresh CDM to an OD_600_ = 0.1 and growth followed for 24 hours with different H_2_O_2_ concentrations in a microplate reader (Infinite M200, Tecan). H_2_O_2_ killing was determined by incubation of bacteria grown from an overnight culture to OD_600_ = 0.3 followed by incubation with 80 mM H_2_0_2_ for 1 or 2 h. MIC determination was performed according to European Committee on Antimicrobial Susceptibility Testing (EUCAST) guidelines using CDM medium (at least three biological replicates for each strain).

### RNA-Seq analysis

Strains were grown in triplicates to OD_600_ = 0.3, split into treated (ATc, 0.1 μg/ml) and untreated control and grown for 30 min, 37°C. Purified RNA was sent to Vertis Biotechnologie AG Freiburg for RNA Sequencing based on Illumina Next Seq 500 system. RNA was examined by a capillary electrophoresis on a Shimadzu MultiNA microchip followed by rRNA depletion using Ribo-Zero rRNA removel Kit from Illumina. RNA was converted to cDNA by fragmenting RNA samples by ultrasound and ligating an oligonucleotide adapter to the 3’end of the RNA. Using M-MLV reverse transcriptase first strand cDNA was created using 3’ adapter as primer. The 5’Illumina TruSeq sequencing adapter was ligated to the 3’end of the purified (Agencourt AMPure XP kit) cDNA and PCR was performed. Samples were pooled in equimolar amounts and fractionated in a size range of 200–500 bp using a preparative agarose gel and Illumina sequencing was performed using 75 bp reads. RNA-Seq analysis was performed using CLC Genomic Workbench (Qiagen). Reads were trimmed (TrueSeq-Antinsense Primer AGATCGGAAGAGCACACGTCTGAACTCCAGTCA) and mapped to the reference genome of HG001 (NZ_CP018205.1). Differential gene expression was performed comparing Rel-Syn or RelQ versus the (pp)pGpp^0^ mutant. Venn diagrams were performed comparing Rel-Syn vs. control and RelQ vs. control. Genes with at least 3-fold difference and a p-value ≥0.001 were defined as differentially regulated compared to the untreated control. Annotation of genes are according to recent “Aureowiki” annotation of strain 8325 (http://aureowiki.med.uni-greifswald.de/Main_Page) [44]. Of the previously identified RNA segments [81] those annotated as indep, s-indep, and indep-NT were regarded as potential regulatory RNAs (sRNAs) and included in the analysis.

## Supporting information

S1 Fig ATc treatment of strains containing empty vector pCG248. Strain HG001 and derivatives were grown to OD_600_ = 0.3 and treated for 30 min with or without 0.1 μg/ml ATc (mutant strains with inducible *rel-Syn*, *relQ* or empty vector). For Northern blot analysis, RNA was hybridized with digoxigenin-labelled probes specific for *sodA*, *ftnA*, *dps*, *per*, *agrA* or *psm*. The 16S rRNA detected in ethidium bromide-stained gels is indicated as a loading control.
(TIF)

S2 Fig *Rel-Syn* induction in HG001 WT. Gene expression following *rel-Syn* induction in HG001 wild type. HG001 was grown to OD_600_ = 0.3 and treated for 30 min without or with 0.1 μg/ml ATc. Transcript were quantified by qRT-PCR on equal amount of total RNA. Statistical significance was determined by two-tailed Student´s T-test, *p ≤ 0.05, **p ≤ 0.01, ***p ≤ 0.001 and ****p ≤ 0.0001
(TIF)

S3 Fig *Rel-Syn* induction in *fur*, *per* and *sar* mutants qRT-PCR results accompanying Fig 5. Quantification of mRNA by qRT-PCR based on three biological replicates. Statistical significance was determined by two-tailed Student´s T-test, *p ≤ 0.05, **p ≤ 0.01, ***p ≤ 0.001 and ****p ≤ 0.0001.
(TIF)

S4 Fig H_2_O_2_ kill curves. Strains were grown to OD_600_ = 0.3 and then treated with 80mM H_2_O_2_ for 1 or 2 h.
(TIF)

S5 Fig H_2_O_2_ does not induce stringent response in *S*. *aureus*. Strains were grown to OD_600_ = 0.3 and treated with mupirocin or H_2_0_2_ for 30 min. RNA was hybridized with digoxigenin-labelled probes specific for *ftnA*, *dps*, *psm*, *agrA* or *rpsl*. The 16S rRNA detected in ethidium bromide-stained gels is indicated as a loading control in the bottom lane.
(TIF)

S6 Fig Elution profile of GTP versus pGpp analyzed using ESI-TOF (micrO-TOF II, Bruker) mass spectrometer connected to an UltiMate 3000 high-performance liquid chromatography analyzed using ESI-TOF (nnnO-TOF II, Bruker) mass spectrometer connected to an NNNMate 3000 high-performance liquid chromatography. (TIF)

S1 Table Strains and plasmids. (DOCX)

S2 Table Oligonucleotides. (DOCX)

S1 Methods Synthesis of guanosine 3'-*O*-diphosphate 5'-*O*-phosphate pGpp. (DOCX)

S1 Data Expression profile of all genes significantly affected upon induction of *rel-Syn* or *relQ*. Role categories and regulons.
(XLSX)

S2 Data Expression profile of all genes upon induction of *rel-Syn* or *relQ*. (XLSX)
