# Supplementary figures and images for: Inducible expression of (pp)pGpp synthetases in Staphylococcus aureus is associated with activation of stress response genes
Source: PLoS Genet. 2020 Dec 30;16(12):e1009282. doi: 10.1371/journal.pgen.1009282 (PMC7802963; doi:10.1371/journal.pgen.1009282)

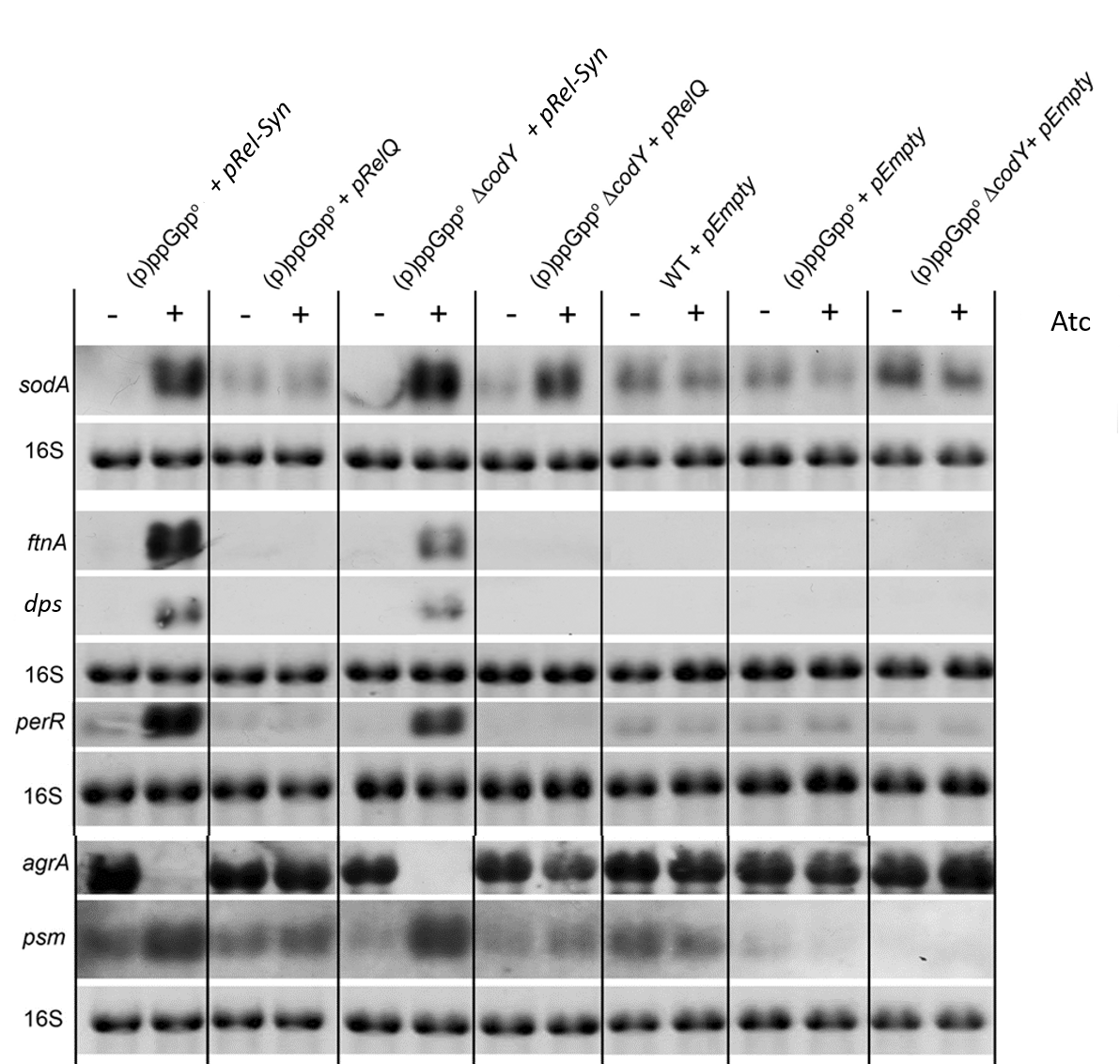

Supplement: S1 Fig — Strain HG001 and derivatives were grown to OD600 = 0.3 and treated for 30 min with or without 0.1 μg/ml ATc (mutant strains with inducible rel-Syn, relQ or empty vector). For Northern blot analysis, RNA was hybridized with digoxigenin-labelled probes specific for sodA, ftnA, dps, per, agrA or psm. The 16S rRNA detected in ethidium bromide-stained gels is indicated as a loading control. (TIF) [file pgen.1009282.s001.tif]

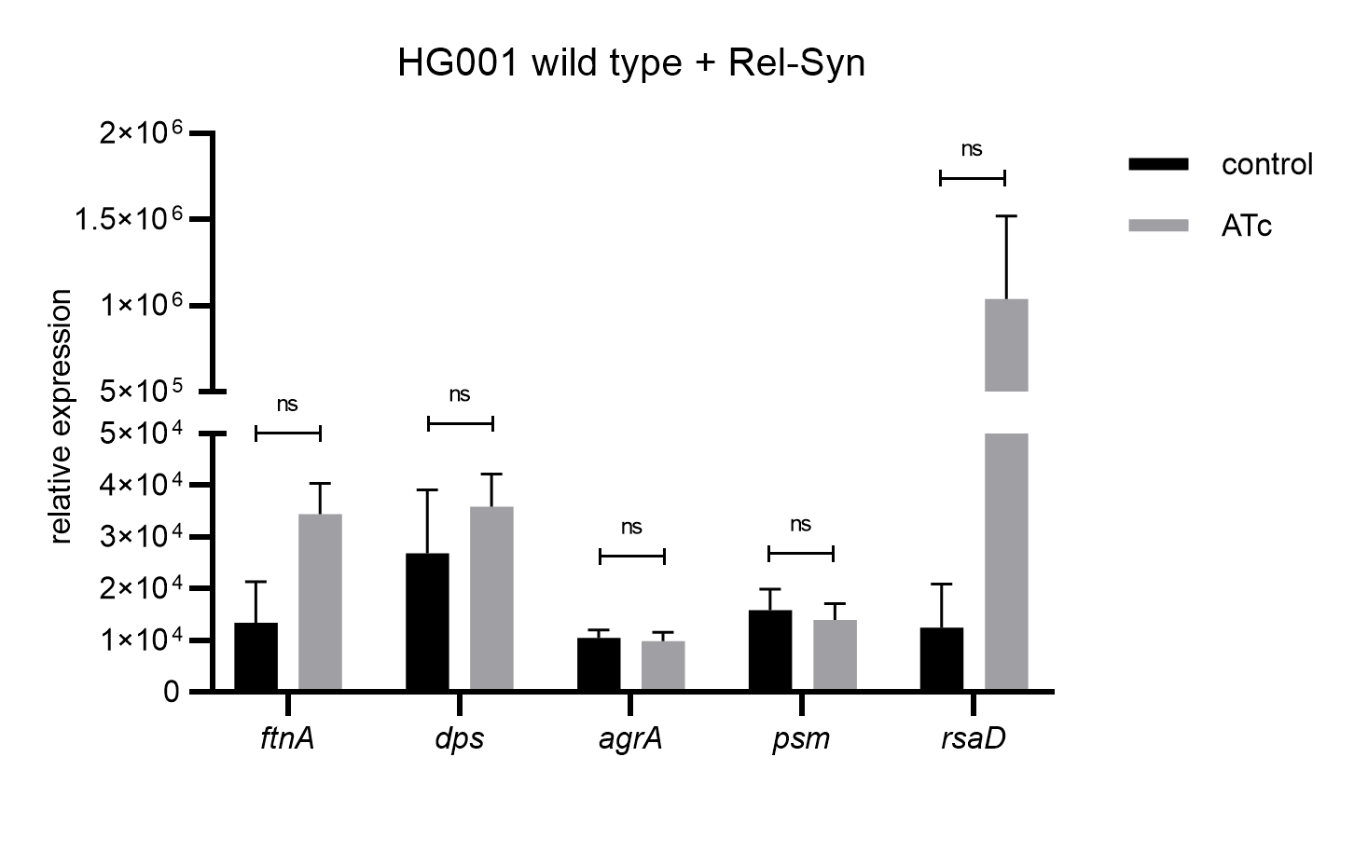

Supplement: S2 Fig — Gene expression following rel-Syn induction in HG001 wild type. HG001 was grown to OD600 = 0.3 and treated for 30 min without or with 0.1 μg/ml ATc. Transcript were quantified by qRT-PCR on equal amount of total RNA. Statistical significance was determined by two-tailed Student´s T-test, *p ≤ 0.05, **p ≤ 0.01, ***p ≤ 0.001 and ****p ≤ 0.0001 (TIF) [file pgen.1009282.s002.tif]

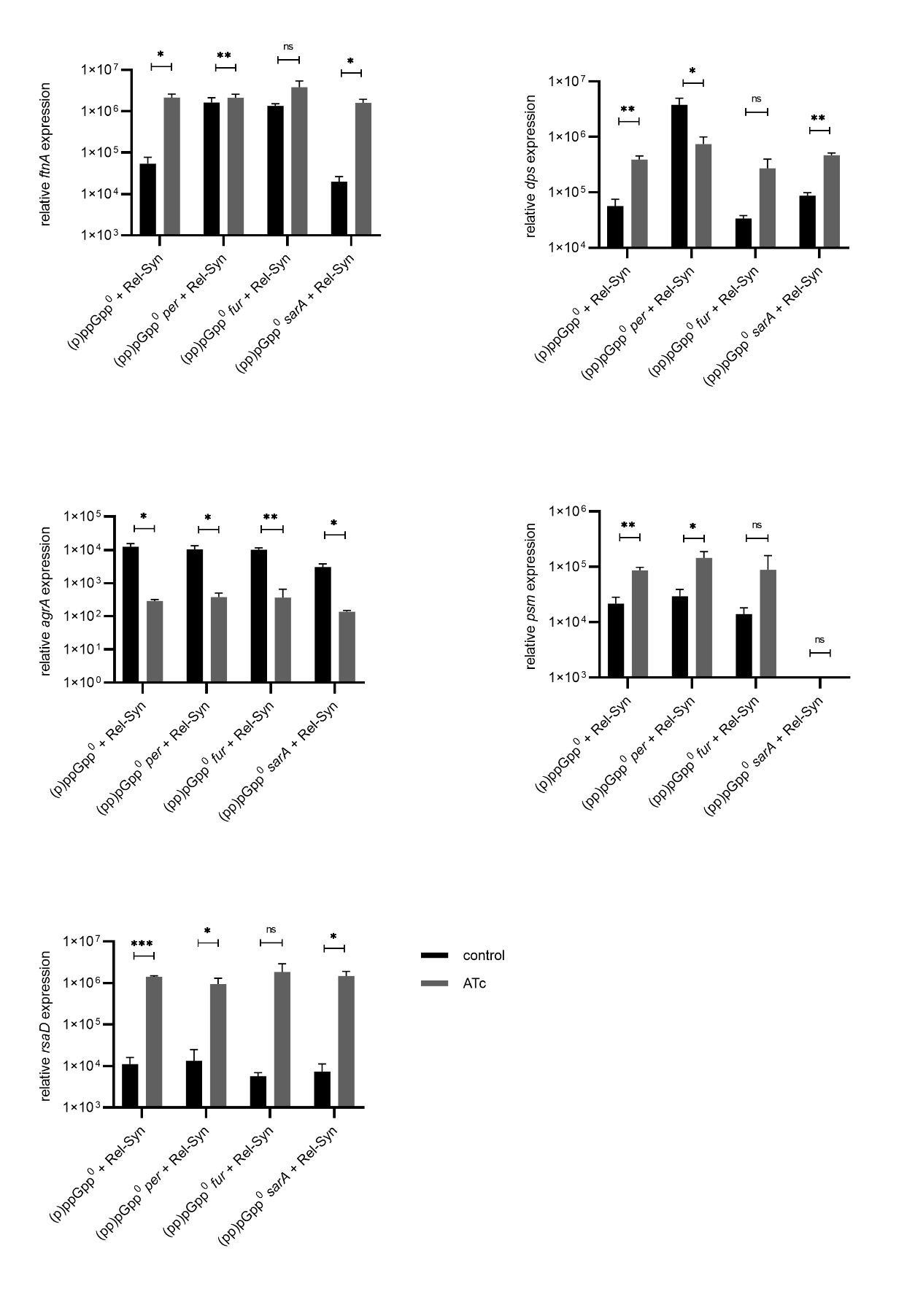

Supplement: S3 Fig — Quantification of mRNA by qRT-PCR based on three biological replicates. Statistical significance was determined by two-tailed Student´s T-test, *p ≤ 0.05, **p ≤ 0.01, ***p ≤ 0.001 and ****p ≤ 0.0001. (TIF) [file pgen.1009282.s003.tif]

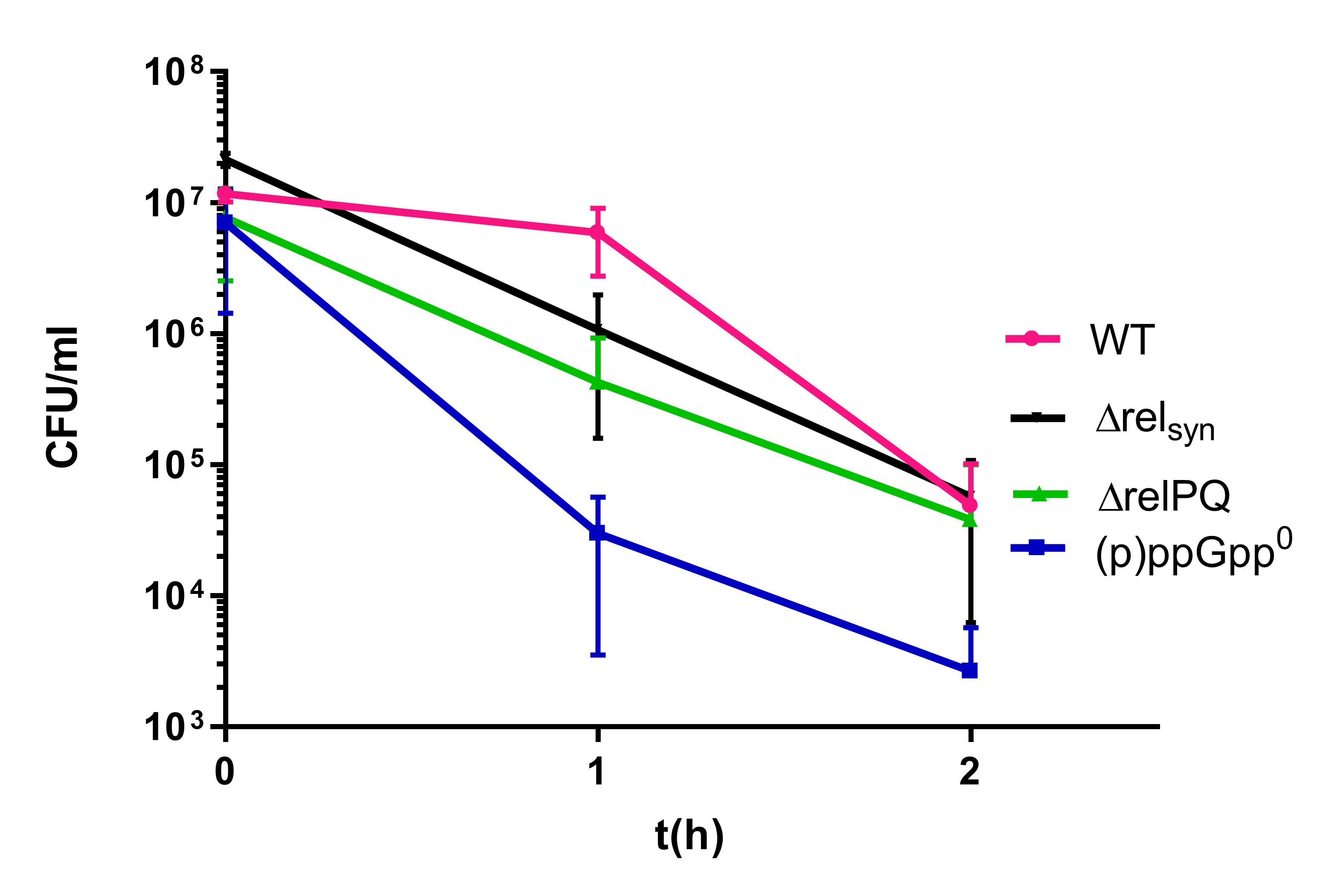

Supplement: S4 Fig — Strains were grown to OD600 = 0.3 and then treated with 80mM H2O2 for 1 or 2 h. (TIF) [file pgen.1009282.s004.tif]

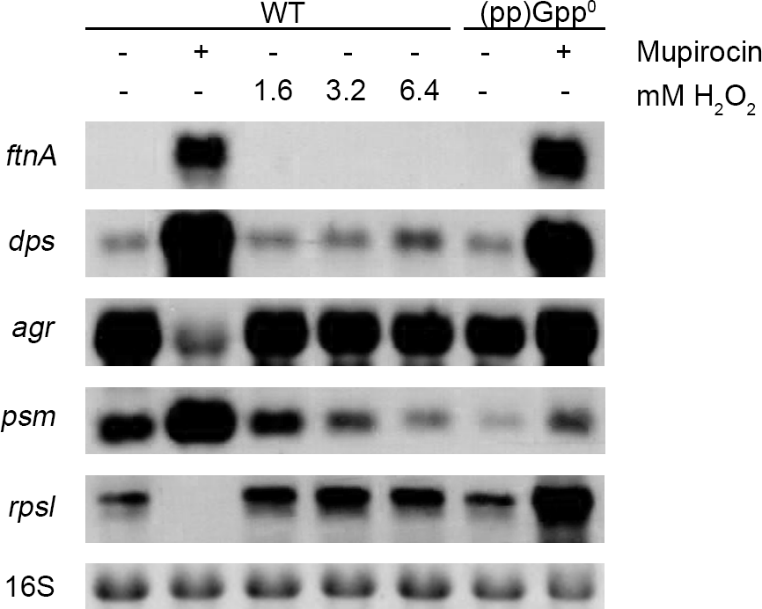

Supplement: S5 Fig — Strains were grown to OD600 = 0.3 and treated with mupirocin or H202 for 30 min. RNA was hybridized with digoxigenin-labelled probes specific for ftnA, dps, psm, agrA or rpsl. The 16S rRNA detected in ethidium bromide-stained gels is indicated as a loading control in the bottom lane. (TIF) [file pgen.1009282.s005.tif]

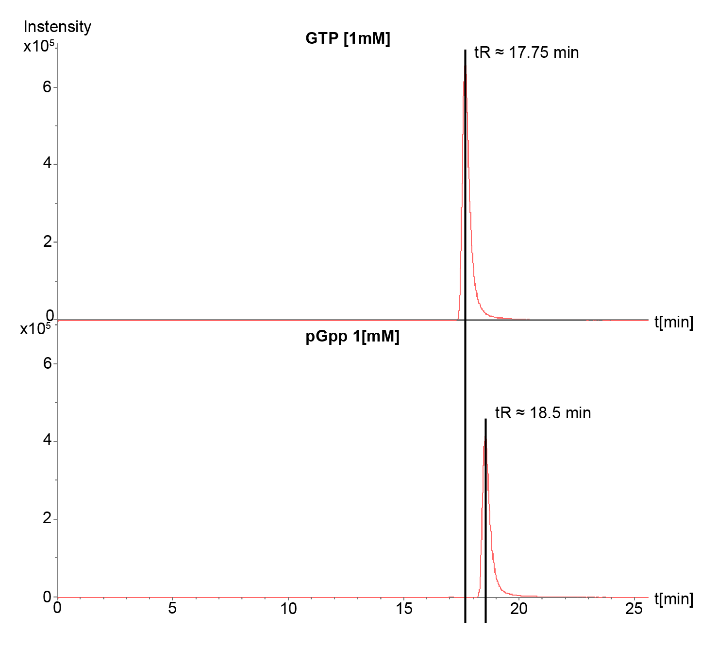

Supplement: S6 Fig — (TIF) [file pgen.1009282.s006.tif]
