# Supplementary material for: Inducible expression of (pp)pGpp synthetases in Staphylococcus aureus is associated with activation of stress response genes
Source: PLoS Genet. 2020 Dec 30;16(12):e1009282. doi: 10.1371/journal.pgen.1009282 (PMC7802963; doi:10.1371/journal.pgen.1009282)
Supplement: S1 Table — (DOCX) [file pgen.1009282.s007.docx]

**Table S1. Stains and plasmids**

| **Strains** | **Description** | **Source/ Reference** |
| --- | --- | --- |
| ***E.Coli*** |  |  |
| BL21 | fhuA2 [lon] ompT gal (λ DE3) [dcm] ΔhsdS λ DE3= λ sBamHlo ΔEcoRI-B int:: (lacl::PlacUV5::T7 gene1) i21 Δnin5, competent for protein expression | NEB |
| ***S.aureus*** |  |  |
| RN4220 | Restriction deficient derivate of 8325-4, rK^-^mK^+^ | [1] |
| Ne1193 | Tnbursa::*sarA erm* | NARSA |
| NE99 | Tnbursa::*fur erm* | NARSA |
| NE665 | Tnbursa::*perR erm* | NARSA |
| HG001 | RN1 derivate, rsbU repaired, tcaR | [2]  [3] |
| HG001-86 | Mutation in the synthetase domain of *rel* | [4] |
| HG001-229-230 | Mutation in the synthetase domain of *relP* and *relQ* (ΔrelP_syn_ ΔrelQ_syn_) | [5] |
| HG001 229-230-263 | Mutation in the synthetase domain of *relP* ,*relQ* and complete deletion of *rel* (ΔrelP_syn_ ΔrelQ_syn_ Δrel) | [5] |
| HG001 *fur* | Tnbursa::*fur erm* | This work |
| HG001 229-230-263 *fur* | Mutation in the synthetase domain of *relP* ,*relQ* and complete deletion of *rel* (ΔrelP_syn_ ΔrelQ_syn_ Δrel) Tnbursa::*fur erm* | This work |
| HG001 *perR* | Tnbursa::*perR erm* | This work |
| HG001 229-230-263 *perR* | Mutation in the synthetase domain of *relP* ,*relQ* and complete deletion of *rel* (ΔrelP_syn_ ΔrelQ_syn_ Δrel) Tnbursa::*perR erm* | This work |
| HG001  *psmα psmβ* | *psmα1-4::tetM, psmβ1-2::ermC* | [6] |
| HG001 229-230-263 *psmα psmβ* | Mutation in the synthetase domain of *relP* ,*relQ* and complete deletion of *rel* (ΔrelP_syn_ ΔrelQ_syn_ Δrel)  *psmα1-4::tetM, psmβ1-2::ermC* | This work |
| USA300 JE2 | USA300 derivative, cured of all plasmids | NARSA |
| USA300 JE2 229-230-263 | Mutation in the synthetase domain of *relP* ,*relQ* and complete deletion of *rel* (ΔrelP_syn_ ΔrelQ_syn_ Δrel) | This work |
| **Plasmids** | **Description** | **Source/ Reference** |
| pET15b | Protein expression vector, ampicillin resistance | Novagen |
| pKOR1 | ATc-inducible mutagenisis vector, chloramphenicol resistance | [7] |
| pCG248 | anhydrotetracyclin (ATc) inducible vector, chloramphenicol resistance | [8]  [9] |
| pCG258 | relP cloned into pCG248 | [5] |
| pCG259 | relQ cloned into pCG248 | [5] |
| pCG327 | N-terminal domain of Rel with hydrolase mutated | [10] |
| pCG229 | pKOR1 with integrated, mutated *relP* | [5] |
| pCG230 | pKOR1 with integrated, mutated *relQ* | [5] |
| pCG263 | pKOR1 with integrated, mutated *rel* | [5] |
| pCG551 | N-terminal domain of Rel with hydrolase mutated cloned into pET15b | [10] |
| pCG121 | relP cloned into pET15b | [5] |
| pCG122 | relQ cloned into pET15b | [5] |

1. Kreiswirth BN, Lofdahl S, Betley MJ, O'Reilly M, Schlievert PM, Bergdoll MS, et al. The toxic shock syndrome exotoxin structural gene is not detectably transmitted by a prophage. Nature. 1983;305(5936):709-12. Epub 1983/10/20. PubMed PMID: 6226876.

2. Pohl K, Francois P, Stenz L, Schlink F, Geiger T, Herbert S, et al. CodY in *Staphylococcus aureus*: a regulatory link between metabolism and virulence gene expression. Journal of bacteriology. 2009;191(9):2953-63. Epub 2009/03/03. doi: 10.1128/JB.01492-08. PubMed PMID: 19251851; PubMed Central PMCID: PMC2681790.

3. Herbert S, Ziebandt AK, Ohlsen K, Schafer T, Hecker M, Albrecht D, et al. Repair of global regulators in *Staphylococcus aureus* 8325 and comparative analysis with other clinical isolates. Infection and immunity. 2010;78(6):2877-89. Epub 2010/03/10. doi: IAI.00088-10 [pii]

10.1128/IAI.00088-10. PubMed PMID: 20212089; PubMed Central PMCID: PMC2876537.

4. Geiger T, Goerke C, Fritz M, Schafer T, Ohlsen K, Liebeke M, et al. Role of the (p)ppGpp synthase RSH, a RelA/SpoT homolog, in stringent response and virulence of *Staphylococcus aureus*. Infection and immunity. 2010;78(5):1873-83. Epub 2010/03/10. doi: 10.1128/IAI.01439-09. PubMed PMID: 20212088; PubMed Central PMCID: PMC2863498.

5. Geiger T, Kastle B, Gratani FL, Goerke C, Wolz C. Two small (p)ppGpp synthases in Staphylococcus aureus mediate tolerance against cell envelope stress conditions. Journal of bacteriology. 2014;196(4):894-902. Epub 2013/12/18. doi: 10.1128/JB.01201-13. PubMed PMID: 24336937; PubMed Central PMCID: PMC3911181.

6. Geiger T, Francois P, Liebeke M, Fraunholz M, Goerke C, Krismer B, et al. The stringent response of *Staphylococcus aureus* and its impact on survival after phagocytosis through the induction of intracellular PSMs expression. PLoS pathogens. 2012;8(11):e1003016. Epub 2012/12/05. doi: 10.1371/journal.ppat.1003016. PubMed PMID: 23209405; PubMed Central PMCID: PMC3510239.

7. Bae T, Schneewind O. Allelic replacement in *Staphylococcus aureus* with inducible counter-selection. Plasmid. 2006;55(1):58-63. Epub 2005/07/30. doi: S0147-619X(05)00053-3 [pii]

10.1016/j.plasmid.2005.05.005. PubMed PMID: 16051359.

8. Helle L, Kull M, Mayer S, Marincola G, Zelder ME, Goerke C, et al. Vectors for improved Tet repressor-dependent gradual gene induction or silencing in Staphylococcus aureus. Microbiology (Reading, England). 2011;157(Pt 12):3314-23. Epub 2011/09/17. doi: 10.1099/mic.0.052548-0. PubMed PMID: 21921101.

9. Schroder W, Goerke C, Wolz C. Opposing effects of aminocoumarins and fluoroquinolones on the SOS response and adaptability in Staphylococcus aureus. The Journal of antimicrobial chemotherapy. 2013;68(3):529-38. Epub 2012/11/22. doi: 10.1093/jac/dks456. PubMed PMID: 23169893.

10. Gratani FL, Horvatek P, Geiger T, Borisova M, Mayer C, Grin I, et al. Regulation of the opposing (p)ppGpp synthetase and hydrolase activities in a bifunctional RelA/SpoT homologue from Staphylococcus aureus. PLoS genetics. 2018;14(7):e1007514. Epub 2018/07/10. doi: 10.1371/journal.pgen.1007514. PubMed PMID: 29985927; PubMed Central PMCID: PMCPMC6053245.

11. Schattenkerk C, Wreesmann CTJ, van der Marel GA, van Boom JH. Synthesis of riboguanosine pentaphosphate ppprGpp (Magic Spot II) via a phosphotriester approach. Nucleic Acids Research. 1985;13(10):3635-49. doi: 10.1093/nar/13.10.3635.
