# Supplementary material for: Inducible expression of (pp)pGpp synthetases in Staphylococcus aureus is associated with activation of stress response genes
Source: PLoS Genet. 2020 Dec 30;16(12):e1009282. doi: 10.1371/journal.pgen.1009282 (PMC7802963; doi:10.1371/journal.pgen.1009282)
Supplement: S2 Table — (DOCX) [file pgen.1009282.s008.docx]

**Table S2: Oligonucleotides**

| **Purpose and Description** | **Template** | **Name** | **Sequence** |
| --- | --- | --- | --- |
| RT-qPCR for *ftnA* expression | ftnA-LCfor  ftnA-LCrev | | GGCAATGGCAGCATACTGTG  ATCTTGACGAGCGATTTCAGA |
| RT-qPCR for *dps* expression | dps-LCfor2  dps-LCrev2 | | CACAAAGCTACACAATTTCCAC  GCCAGCATTACCAGCAATTTC |
| RT-qPCR for *agrA* expression | agrA-LCfor  agrA-LCrev | | ACGAGTCACAGTGAACTTAC  GACGGTTATCTAAATGGGCA |
| RT-qPCR for *psmα* expression | psm2391  psm2715 | | CATCGTTTTGTCCTCCTG  TCATCGCTGGCATCATTA |
| RT-qPCR for *rsaD* expression | rsaD-LCfor  rsaD-LCrev | | GGTAATACACTTGGCTTTTATGGG  AGAAGTTATCTCCTTTGTGTTG |
| RT-qPCR for *rpsl* expression | rpslfor  rpslseqrev4 | | ACCACAAAAACGTGGTGTATGTACT  ACCAGGGATGTATGCGTT |
| verification of *relP* synthase mutant | USA300-229-230-263 | relPDIG-for  relPDIG-rev | GTCGCACATTCTTTCAGT  CGTTATTAGGTTTCGTAGAGTT |
| verification of *relQ* synthase mutant | USA300-229-230-263 | relQDIGfor2  relQDIGrev2 | TTCGTAACACTAAAGAAAGTGG  GCGTGTAATATTTTTGAGCT |
| verification of *rsh* mutant | USA300-229-230-263 | rel431for  relLC4rev | GCGTGGCTTTATCATTGG  ACTTCAACCATCATTCGG |
| Verification of *perR* mutant | HG001 *perR*  HG001 229-230-263 *perR* | perR-for  TnUpstream | TGAACTAGAAGAATCAATTGCATCA  CTCGATTCTATTAACAAGGG |
| Verification of *fur* mutant | HG001 *fur*  HG001 229-230-263 *fur* | furtnfor  TnBuster | GCACGTTTCACACACACCAT  GCTTTTTCTAAATGTTTTTTAAGTAAATCAAGTAC |
| Verification of *sarA* mutant | HG001 *sarA*  HG001 229-230-263 *sarA* | sarAtnfor  TnUpstream | GTTGTTTGCTTCAGTGATTCGT  CTCGATTCTATTAACAAGGG |
| Verification of *psmα/β* mutant | HG001 229-230-263 *psm* | | ([Geiger et al., 2012](#_ENREF_63)) |
| Creation of dig-labeled probe *ftnA* | WT HG001 | ftnADig-for  ftnADig-rev | GAGTACTTTGCAGCACACGC  CATTGCTGTCATCGCCGATAC |
| Creation of dig-labeled probe *dps* | WT HG001 | \| mrgADig-for \| \| --- \| \| mrgADig-rev \| | \| GCTACACAATTTCCACTGGT \| \| --- \| \| CATACCTATAAACATATCTTC \| |
| Creation of dig-labeled probe *psm*/*agr* | [6] | | |
